# Itk is required for Th9 differentiation via TCR-mediated induction of IL-2 and IRF4

**DOI:** 10.1038/ncomms10857

**Published:** 2016-03-03

**Authors:** Julio Gomez-Rodriguez, Françoise Meylan, Robin Handon, Erika T. Hayes, Stacie M. Anderson, Martha R. Kirby, Richard M. Siegel, Pamela L. Schwartzberg

**Affiliations:** 1 National Human Genome Research Institute, National Institutes of Health, 49 Convent Drive, Bethesda, Maryland 20892, USA; 2 National Institute of Arthritis and Musculoskeletal and Skin Diseases, National Institutes of Health, 9000 Rockville Pike, Bethesda, Maryland 20892, USA

## Abstract

Th9 cells produce interleukin (IL)-9, a cytokine implicated in allergic asthma and autoimmunity. Here we show that Itk, a mediator of T cell receptor signalling required for Th2 immune responses and the development of asthma, is a positive regulator of Th9 differentiation. In a model of allergic lung disease, Itk-deficient mice show reduced pulmonary inflammation and IL-9 production by T cells and innate lymphoid type 2 cells (ILC2), despite normal early induction of ILC2s. *In vitro*, *Itk*^*−/−*^ CD4^+^ T cells do not produce IL-9 and have reduced levels of IRF4 (Interferon Regulator Factor 4), a critical transcription factor for effector T cell function. Both IL-9 and IRF4 expression are rescued by either IL-2 or constitutively active STAT5, but not NFATc1. STAT5 binds the *Irf4* promoter, demonstrating one mechanism by which IL-2 rescues weakly activated T cells. Itk inhibition also reduces IL-9 expression by human T cells, implicating ITK as a key regulator of Th9 induction.

## Abstract (web summary)

The Tec family tyrosine kinase, Itk, is a component of the T-cell receptor essential for optimal Th2 responses in vivo. Here the authors show in human cells and mouse models that Itk is also needed for the production of IL-9, an important contributor to allergic asthma.

## Graphical abstract

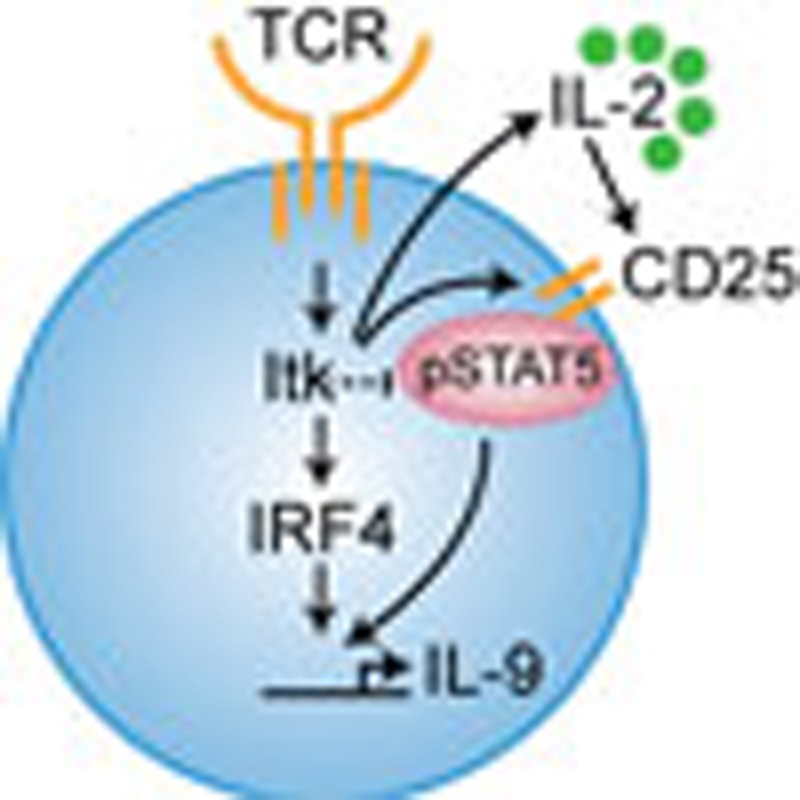

The adaptive immune system plays an important role in specific responses against pathogens. This function is achieved, in part, by the capacity of naive CD4^+^ T cells to differentiate into distinct effector T-helper (Th) subsets upon stimulation through the T-cell receptor (TCR) and co-stimulatory molecules, as well as cytokines secreted by innate immune cells. These CD4^+^ T-cell subsets include Th1, Th2 and Th17 cells that secrete signature cytokines, and regulatory T (Treg) cells that help hold immune responses in check. Th9 cells are a distinct subset of effector CD4^+^ T cells that secrete IL-9 (refs 1, 2). Like IL-4, IL-9 is associated with type II immune responses, such as in allergic asthma. IL-9 is produced both by T cells and by type II innate lymphoid cells (ILC2), which influence the expansion and cytokine production of each other^1^,^2^. IL-9 induces mast cell proliferation, goblet cell hyperplasia, airway hyper-reactivity and IL-13 production; increased IL-9 has been detected in patients with asthma^3^,^4^,^5^,^6^ and in mouse asthma models^7^,^8^,^9^,^10^,^11^,^12^. Furthermore, transgenic expression of IL-9 has been shown to result in allergic inflammation and IL-9 can induce other cytokines and factors involved in allergic hypersensitivity^13^,^14^,^15^. IL-9 also has important roles in the eradication of type-II-inducing pathogens such as *Leishmania major*, *Trichuris muris*, *Schistosoma mansoni* and *Nippostrongylus brasiliensis*^1^.

Th9 cells can be generated from naive CD4^+^ T cells *in vitro* by culturing with IL-4 and TGFβ1 (Transforming growth factor beta 1) (refs 16, 17, 18, 19). In addition, a number of mouse and human studies have identified molecules that potentiate the differentiation of IL-9-producing cells, including the TNF family members OX40 ligand and TNF-like factor 1A (TL1A)^1^,^2^. Interestingly, Th2 and Th9 cells, which are both involved in type II immunity, require IL-4 and similar transcription factors, including STAT6, GATA-3, IRF4, BATF, PU.1 and STAT5, for their differentiation^1^,^2^.

Itk is a member of the Tec family of cytosolic tyrosine kinases and is an important component of TCR-mediated signalling^20^. However, unlike more proximal molecules in TCR signalling, the loss of Itk does not prevent, but rather alters, T-cell activation by modulating the strength or duration of TCR signals^21^,^22^. Cells deficient in Itk have impaired TCR signalling associated with decreased activation of PLC-γ and the downstream pathways involved in Ca^2+^ mobilization, nuclear factor of activated T cell (NFAT) activation and expression, Ras and Erk kinase activation, as well as regulation of the actin cytoskeleton. In CD8^+^ cells, Itk has also been shown to affect expression of the transcription factor IRF4 (ref. 23), expression of which has been described as linking the strength of TCR signals to downstream changes required for effector CD8^+^ T-cell differentiation^24^,^25^. In previous studies, we have shown that Itk has an important role in balancing the differentiation of Th17 and Treg cells. Itk-deficient T cells produce less IL-17A under Th17 conditions^26^ and instead develop higher percentages of FoxP3^+^ cells under both Th17 and Treg-inducing conditions, associated with altered IL-2 signalling with increased STAT5 phosphorylation, yet impaired activation of mTOR pathways^27^. However, some of the first phenotypes identified in Itk-deficient mice were defective Th2 responses, which were seen in response to both parasitic challenges and an ovalbumin inhalation model of allergic asthma^28^,^29^,^30^. Polymorphisms in the *ITK* promoter that increase ITK expression in humans have also been linked to increased asthma incidence^31^. Nonetheless, the effects of Itk deficiency on the differentiation of IL-4 producing Th2 cells *in vitro* are less clear^32^, suggesting that other aspects of type II immunity may be affected by the loss of Itk.

Here we analyse the contribution of Itk to Th9 cell differentiation. When differentiated under Th9 conditions in culture, Itk-deficient CD4^+^ T cells fail to express the Th9 signature cytokine IL-9. This defect is one of the most profound phenotypes observed in *Itk*^*−/−*^ cells to date and is associated with reduced levels of IRF4, pS6 and pSTAT5, all of which can be rescued by inclusion of IL-2 under Th9 conditions. Chromatin immunoprecipitation (ChIP) analyses reveal STAT5 binds the *Irf4* promoter, but not in the absence of Itk, directly implicating IL-2 in the induction of *Irf4*, which is required for Th9 differentiation, and supporting the idea that IL-2 is a critical cytokine that potentiates T-cell activation under conditions of weak stimulation. Furthermore, in a model of papain hypersensitization that elicits a strong Th9 response, Itk-deficient mice have decreased pathology and reduced numbers of both IL-9-producing T cells and ILC2s, despite normal early ILC2 induction. Finally, decreased IL-9 is also detected in human CD4^+^ T cells treated with an Itk inhibitor, suggesting a possible approach to prevent Th9-mediated disease.

## Results

### Itk is essential for IL-9 expression by T cells in culture

**Figure 1:**
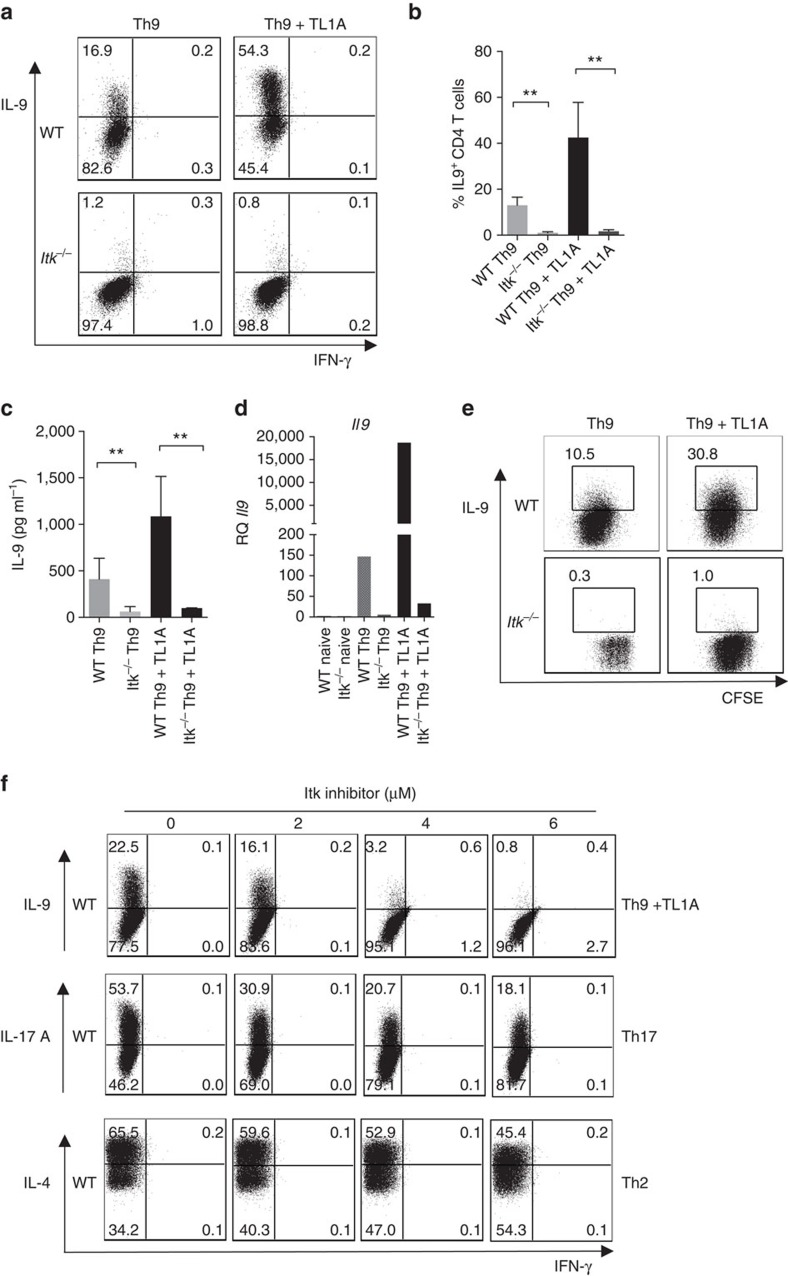
Itk is required for Th9 differentiation. (**a**–**d**) Sorted naive CD4^+^ T cells from WT and *Itk*^*−/−*^ mice were differentiated under Th9 conditions (1 μg ml^−1^ anti-CD3, 3 μg ml^−1^ anti-CD28, 20 ng ml^−1^ IL-4, 5 ng ml^−1^ TGFβ1 with or without 10 ng ml^−1^ TL1A, in presence of T-depleted splenocytes as APCs) for 3 days, (**a**) cells were restimulated with PMA and Ionomycin and IL-9 and IFN-γ production were analysed by intracellular staining. Representative flow plots from one out of 10 experiments. (**b**) Mean±s.e.m. of 10 independent experiments **P<0.01, using two-tailed unpaired Student's *t*-test. (**c**) IL-9 was determined by Luminex in supernatants from cells differentiated as in **a**, before restimulation. Mean±s.e.m of three independent experiments are shown, **P<0.01, using two-tailed unpaired Student's *t*-test. (**d**) mRNA of *Il9* of cells differentiated as in **a** were determined by qRT-PCR. (**e**) Sorted naive CD4^+^ T cells were stained with CSFE, differentiated and stained as in **a**. (**f**) Sorted naïve CD4^+^ T cells from WT and *Itk*^*−/−*^ mice were differentiated under Th9 conditions plus TL1A, Th17 or Th2 conditions for 3 days in presence of increasing concentrations of Itk inhibitor BMS-509744 as indicated in the figure. Cells were restimulated with PMA and Ionomycin and stained for IL-9, IL-17A, IL-4 and IFN-γ. Results in panels **d,e** and **f** are representative from one of three independent experiments.

Previous studies have shown that Itk-deficient mice are resistant to Th2-induced lung hypersensitivity in response to allergens or parasites, with decreased Th2 cytokines detected in the lung^29^,^30^. Nonetheless, we find that naive Itk-deficient T cells can differentiate into IL-4 producing cells *in vitro* (Supplementary Fig. 1a), raising the question of whether Itk may affect expression of other cytokines implicated in asthma. To examine the effects of Itk on IL-9 expression, naive CD4^+^ T cells from wild-type (WT) and Itk-deficient mice were activated *in vitro* with anti-TCR and CD28 antibodies plus WT T-depleted splenocytes as antigen presenting cells (APCs) in the presence of IL-4 and TGFβ1, conditions that mediate naive CD4^+^ T-cell differentiation into IL-9-producing effector cells^16^,^17^,^18^,^19^. In some cultures, we also included the TNF family cytokine TL1A, which enhances the generation of IL-9-producing cells^33^. Intracellular staining revealed that under standard Th9 conditions (IL-4 plus TGFβ1), there was a dramatic reduction in the production of IL-9 from Itk-deficient cells, with very few mutant CD4^+^ T cells producing IL-9 (0.95±0.3%) compared with WT cells (13±1.7%) (Fig. 1a,b). Surprisingly, even in presence of TL1A, *Itk*^*−/−*^ cells barely increased the percentage of IL-9^+^ cells (1.7±0.3% compared with 42.5±7.6% for WT cells; Fig. 1b). Secreted IL-9 was also markedly reduced in *Itk*^*−/−*^ cultures compared with WT (Fig. 1c). This defect occurred at the level of mRNA, as that *Itk*^*−/−*^ cells exhibited very poor induction of *Il9* message (Fig. 1d). Nonetheless, Itk-deficient cells still showed similar levels of *Il4r* and pSTAT6, as well as increased expression of *Tgfβr1* (Supplementary Fig. 1b–d).

To evaluate proliferation in Th9 cultures, we stained naive CD4^+^ T cells with carboxyfluorescein succinimidyl ester (CFSE) to track cell division. *Itk*^*−/−*^cells showed reduced division as previously reported^34^,^35^,^36^, in addition to decreased IL-9 production compared with WT cells (Fig. 1e). However, although TL1A increased cell division in Itk-deficient cells (Supplementary Fig. 1e), even cells that had undergone equivalent divisions to WT cells, remained defective for IL-9 production.

T cells from Itk-deficient mice undergo altered development, with increased numbers of activated cytokine-secreting innate type lymphocytes^37^,^38^,^39^. Although we used sorted naive cells in our experiments, to further rule out effects of altered development, we differentiated naive CD4^+^ T cells from WT mice with increasing concentrations of an Itk inhibitor, BMS-509744, which is known to inhibit PLC-γ1 phosphorylation, a downstream readout of Itk activity^40^. Treatment of WT cells with the Itk inhibitor led to a dose-dependent reduction in IL-9-producing cells, with maximal inhibition reached at 6 uM (0.8% IL-9^+^ at 6 uM compared with 22.5% without the inhibitor; Fig. 1f). Similar results were seen with another inhibitor, 10N 23 (data not shown). The Itk inhibitor also reduced IL-17A production from WT naive CD4^+^ T cells under Th17 conditions, although this reduction was only partial (18.1% at 6 μM compared with 53.7% without the inhibitor; Fig. 1f), resembling the defect in IL-17A production in Itk-deficient T cells (Supplementary Fig. 1a, lower panel and ref. 26). Similarly, we observed only a partial defect in IL-4 production in cells stimulated under Th2 conditions in the presence of the Itk inhibitor (Fig. 1f). Thus, using the same inhibitor concentrations, Itk-deficient cells still produced some cytokines under Th17 and Th2 conditions, while they produced virtually no IL-9 under Th9 conditions. These results confirm that Itk is a positive modulator of IL-9 expression that is required for the generation of Th9 cells.

### IL-9 induction correlates with TCR signalling

**Figure 2:**
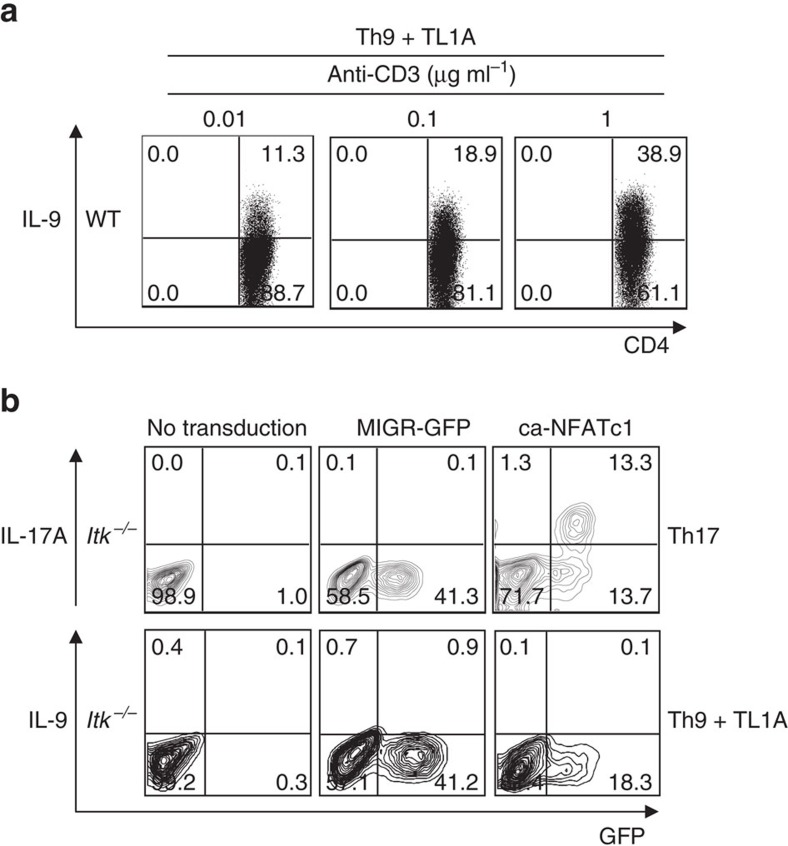
IL-9 expression correlates with strength of TCR signals. (**a**) Sorted naive WT CD4^+^ T cells were differentiated under Th9 conditions plus TL1A with 0.01, 0.1 or 1 μg ml^−1^ of anti-CD3, then restimulated with PMA and Ionomycin and IL-9 production analysed by intracellular staining. (**b**) Itk-deficient CD4^+^ T cells were transduced with retroviruses expressing constitutively active NFATc1 (ca-NFATc1), or a control (MIGR), differentiated under Th9 plus TL1A or Th17 conditions, and cytokine production determined by intracellular staining after PMA and ionomycin restimulation. Results in **a** and **b** are from one representative of three independent experiments.

We and others have previously suggested that Itk acts as a modulator of TCR signalling, required for full activation of T cells^22^,^41^. To evaluate how TCR signals influence the induction of Th9 responses, we differentiated naive WT CD4^+^ T cells under Th9 conditions in presence of increasing concentrations of anti-CD3. We observed a direct correlation between the expression of IL-9 and the stimulating concentration of anti-CD3 (α-CD3), detecting 11.3% and 38.9% of T cells expressing IL-9 with 0.01 and 1 μg ml^−1^ of anti-CD3, respectively (Fig. 2a). Thus, IL-9 expression directly correlates with the extent of TCR engagement, suggesting a link between the impaired IL-9 production in Itk-deficient cells and their defects in TCR signalling.

TCR-mediated Ca^2+^ mobilization activates the NFATs transcription factors, which are dephosphorylated by calcineurin, leading to their nuclear localization^42^. Treatment of WT cells with a calcineurin inhibitor decreased *Il9* expression, demonstrating a role for this pathway in the regulation of *Il9* (data not shown). Itk is required for both full activation and induction of NFAT in response to TCR activation^28^,^29^; expression of an activated NFATc1 mutant (ca-NFATc1) rescues expression of IL-17A, another calcineurin-sensitive cytokine, in Itk-deficient cells (Fig. 2b and ref. 26). Nonetheless, although transduction of a ca-NFATc1 construct increased expression of IL-9 in WT cells (data not shown), it failed to rescue IL-9 expression in *Itk*^*−/−*^ cells (Fig. 2b, lower panel). Thus, NFATc1 was not sufficient to rescue the defect in Th9 differentiation in Itk-deficient CD4^+^ T cells.

### Itk is required for IRF4 expression

**Figure 3:**
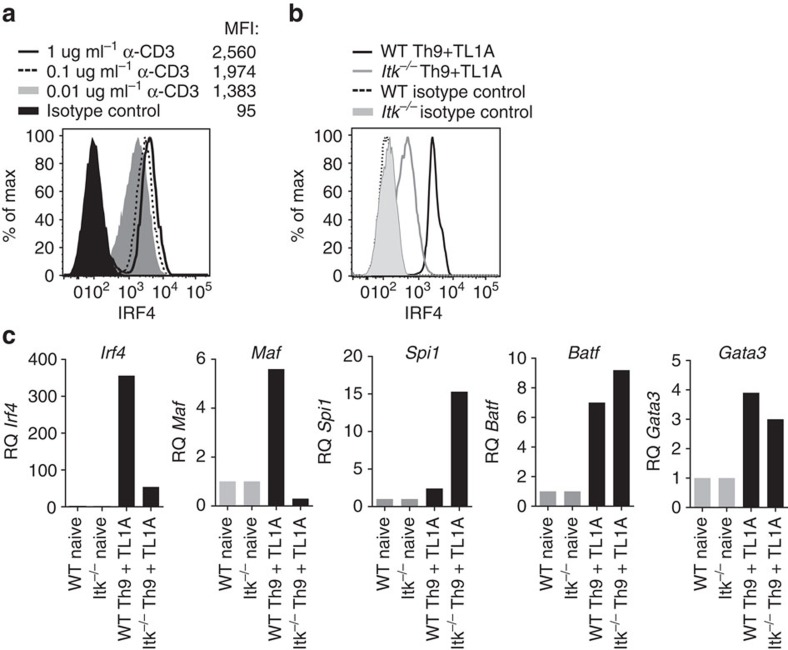
Itk is required for IRF4 expression in CD4^+^ T cells. (**a**) Sorted naive WT CD4^+^ T cells were differentiated under Th9 plus TL1A conditions, with 0.01, 0.1 or 1 μg ml^−1^ of anti-CD3, and then stained for intracellular IRF4 (MFI is indicated). (**b**) Intracellular levels of IRF4 from WT and *Itk*^*−/−*^ CD4^+^ T cells differentiated under Th9 plus TL1A for 3 days. (**c**) mRNAs levels of *Irf4* and *Maf* or *Spi1*, *Batf* and *Gata3* were determined by qRT-PCR on cells differentiated as in **b**. Data in **a**-**c** are representative of one of at least three independent experiments.

To understand potential mechanisms by which Itk affects Th9 differentiation, we examined other transcription factors expressed in Th9 cells, including IRF4, BATF, PU.1, MAF and GATA-3. IRF4 was of particular interest as that it is required for Th9 differentiation, is induced in response to increased TCR signalling in CD8^+^ T cells^24^ and exhibits decreased induction in CD8^+^ T cells from Itk-deficient mice^23^. Consistent with these results, we saw that IRF4 expression correlated with the extent of TCR stimulation in WT CD4^+^ T cells, with a MFI (mean fluorescence intensity) of 2,560 at 1 μg ml^−1^ of anti-CD3 versus 1,383 with 0.01 μg ml^−1^ anti-CD3 (Fig. 3a). Furthermore, we observed that *Itk*^*−/−*^ CD4^+^ T cells polarized under Th9 conditions exhibited marked reductions of IRF4 protein and mRNA, compared with WT cells (Fig. 3b,c). Itk-deficient cells also showed decreased expression of *Maf* (Fig. 3c), which encodes a transcription factor expressed in Th9 cells, although its requirement for Th9 differentiation is less clear^17^. In contrast, expression of *Spi1*, encoding PU.1, which may be upregulated in response to TGFβ1 (ref. 8) was actually increased in Itk-deficient cells (as was *Tgfβr1*), while expression of BATF and GATA-3 were only mildly affected. Thus, Itk-mediated pathways contribute to the induction of IRF4, a critical transcription factor for Th9 differentiation^19^.

### IL-2 rescues IL-9 production in Itk-deficient CD4^+^ T cells

**Figure 4:**
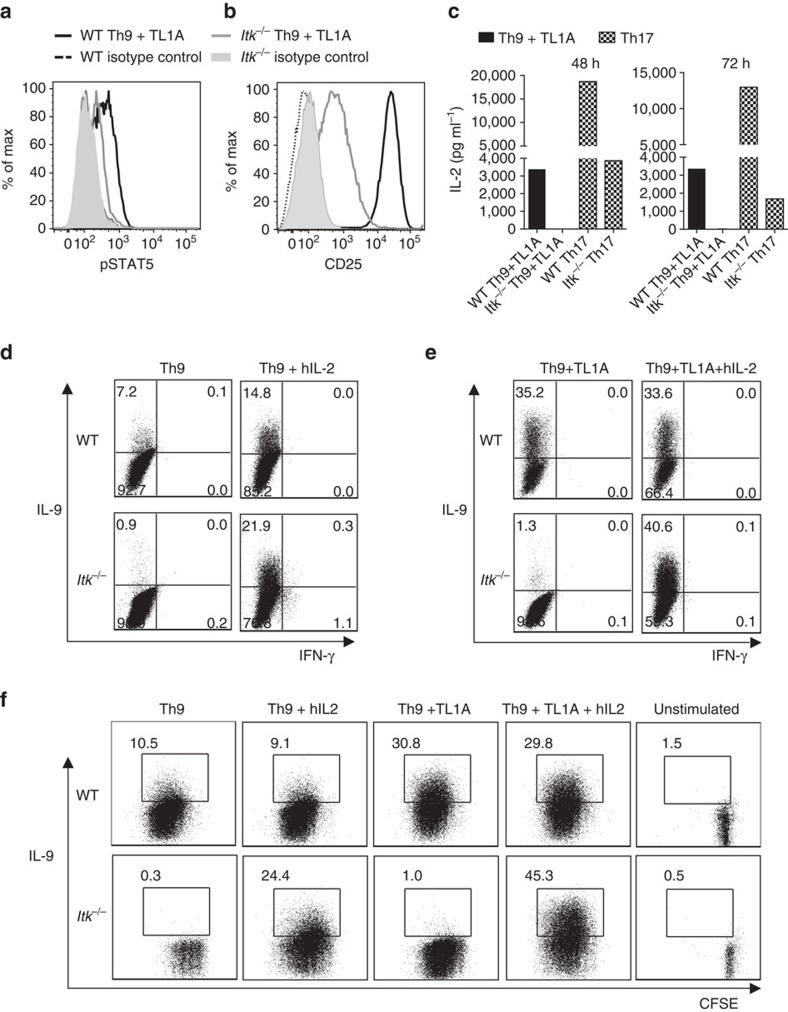
IL-2 rescues Th9 differentiation in *Itk*^*−/−*^ **CD4**^**+**^ **T cells.** (**a**-**c**) Sorted naïve CD4^+^ T cells from WT and *Itk*^*−/−*^ mice were differentiated under Th9 conditions plus TL1A for 3 days and pSTAT5 (**a**) and CD25 (**b**) were determined by flow cytometry: WT (black), *Itk*^*−/−*^ (grey) lines. (**c**) Sorted naive CD4^+^ T cells were differentiated as in **a** or under Th17 conditions and IL-2 in supernatants were determined at 48 and 72 h by Luminex. Th9 conditions: black bars. Th17 conditions: hatched bars. (**d**,**e**) Sorted naïve CD4^+^ T cells were differentiated for 3 days under Th9 (**d**) or Th9 plus TL1A (**e**) conditions in absence or presence of blocking anti-IL-2 plus hIL-2, restimulated with PMA and Ionomycin and IL-9 analysed by flow cytometry. (**f**) Sorted naive CD4^+^ T cells from WT and *Itk*^*−/−*^ mice were stained with CSFE, differentiated, and restimulated with PMA and Ionomycin to evaluate IL-9 expression. Data in **a**-**f** are representative of one out of at least three independent experiments.

One of the other transcription factors we evaluated was STAT5, which is phosphorylated downstream of IL-2 and binds the *Il9* promoter^33^,^43^,^44^. IL-2 and STAT5 have been shown to play critical roles in the expression of IL-9 (ref. 45). Notably, *Itk*^*−/−*^ Th9 cells exhibited less phosphorylated STAT5 (pSTAT5) compared with WT Th9 cells after 72 h of differentiation (Fig. 4a); similar results were also obtained after 24 h (Supplementary Fig. 2a). These results were somewhat surprising as that we have observed that Itk-deficient cells show increased pSTAT5 under multiple conditions including Th17 and Treg-inducing cultures^27^. *Itk*^*−/−*^ cells also showed a defect in the expression of the high affinity IL-2 receptor α (CD25), the induction of which is regulated both by TCR and IL-2 (Fig. 4b). These results suggested that IL-2 signals are reduced in *Itk*^*−/−*^ cells during Th9 differentiation.

To further evaluate these observations, we analysed the levels of IL-2 in supernatants during Th9 differentiation. We found that Itk-deficient cells had undetectable levels of IL-2 in Th9 culture supernatants at 24, 48 and 72 h (Fig. 4c; Supplementary Fig. 2b). Although Itk deficiency has been shown to impair IL-2 expression^34^,^35^,^36^, this defect appeared to be particularly severe under Th9 differentiation conditions; *Itk*^*−/−*^ cells were capable of secreting at least some IL-2 during Th17 differentiation (Fig. 4c). Of note, overall our Th17 conditions also gave much higher levels of IL-2 in the media than those observed in Th9 cultures.

To further dissect the contribution of IL-2 to defects in *Itk*^*−/−*^ cells during Th9 differentiation, we used anti-mouse IL-2 to eliminate differential contributions of autocrine IL-2 production, and added exogenous human IL-2, which can interact with and stimulate the murine IL-2 receptor. Addition of human IL-2 to Th9 cultures of naive CD4^+^ T from WT and *Itk*^*−/−*^ mice led to a dramatic increase of IL-9 production in *Itk*^*−/−*^ cells, even above that seen in WT cells (from 0.75±0.25 to 19.87±5.5 in the presence of hIL-2, compared with 10.3±3.6 for WT cells), (Fig. 4d; Supplementary Fig. 3a), consistent with previous data showing increased responsiveness to IL-2 in the absence of Itk or under weak TCR stimulation conditions^27^,^46^,^47^. Similarly, IL-2 also rescued IL-9 production in Itk-deficient cells stimulated in the presence of TL1A (increasing from 1.6±1.06% without hIL-2 to 40.82±13.3% in the presence of hIL-2) (Fig. 4e; Supplementary Fig. 3b). IL-2 also increased proliferation in Itk-deficient cells, although increased IL-9 expression was observed even in cells that still had not proliferated as well as WT cells (see Th9 plus hIL-2, Fig. 4f). Consistent with the rescue by exogenous IL-2, the lack of IL-9 expression in Itk-deficient T cells could also be rescued by co-culture with WT (CD45.1) CD4^+^ T cells (Supplementary Fig. 3c). Moreover, re-expression of Itk in *Itk*^*−/−*^ cells by retroviral transduction rescued IL-9 expression in both the GFP^+^ (transduced) and the GFP^-^ (untransduced) cells (Supplementary Fig. 3d), consistent with both cell autonomous and non-autonomous effects, likely as a result of IL-2 produced by the transduced cells re-expressing Itk. Thus, the lack of IL-9 expression in *Itk*^*−/−*^ cells can be rescued by exogenous IL-2.

**Figure 5:**
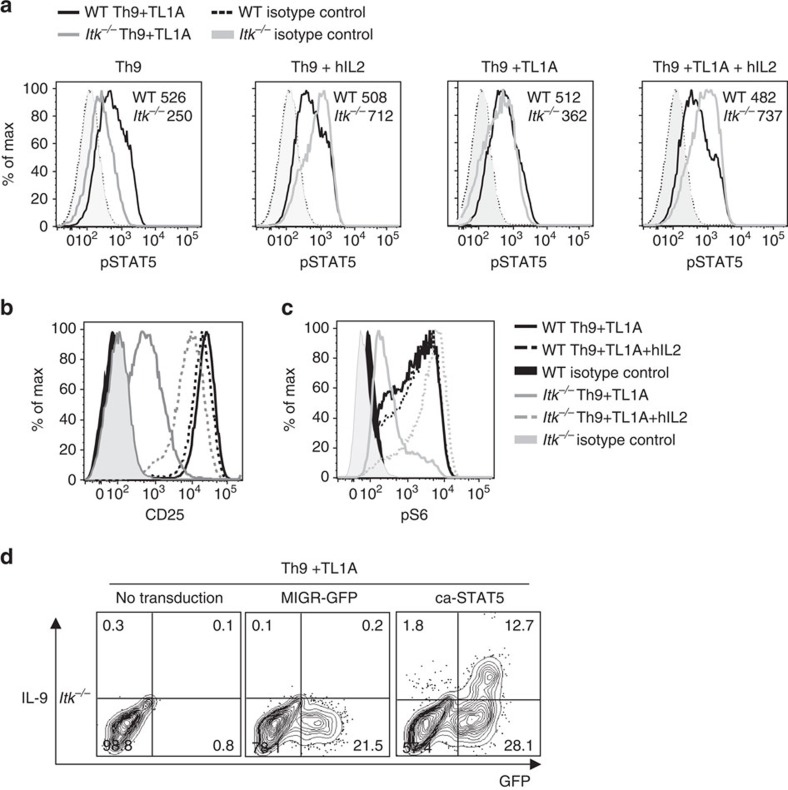
IL-2 rescues pSTAT5 and pS6 in *Itk*^*−/−*^ CD4 T cells. (**a**-**c**) Sorted naive CD4^+^ T cells from WT and *Itk*^*−/−*^ mice were differentiated for 3 days under Th9 or Th9 plus TL1A conditions in absence or presence of blocking anti-IL-2 plus hIL-2 and analysed for pSTAT5 (MFI are indicated) (**a**) CD25 (**b**) and pS6 (**c**). (**d**) Itk-deficient CD4^+^ T cells were transduced with control (MIGR) or constitutively active STAT5-expressing retroviruses, differentiated under Th9 plus TL1A conditions and IL-9 production determined by intracellular staining after PMA and Ionomycin restimulation. Results in **a**-**d** are representative of one out of at least 3 experiments.

Increased responsiveness to IL-2 in the absence of Itk was also reflected at the level of pSTAT5 (Fig. 5a). Consistent with the increased responses to IL-2, we also saw increased expression of CD25 after inclusion of IL-2 (Fig. 5b). Another pathway downstream of both TCR and IL-2 that is impaired in Itk-deficient cells is the activation of mTORC1, which can be monitored by the phosphorylation of S6, a downstream substrate of the mTORC1 target S6 kinase. Again, inclusion of hIL-2 rescued phosphorylation of S6, even above that observed in WT cells (Fig. 5c).

Finally, to determine whether the impaired IL-9 expression in *Itk*^*−/−*^ cells could be rescued by STAT5 activation, *Itk*^*−/−*^ cells were stimulated under Th9 conditions and transduced with a retrovirus expressing constitutively activated STAT5 (ca-STAT5). Expression of ca-STAT5 greatly improved IL-9 expression in *Itk*^*−/−*^cells, increasing it from 0.1 to 12.7% (Fig. 5d), supporting the idea that impaired STAT5 activation contributes to the defect in IL-9 expression in *Itk*^*−/−*^ cells.

### IL-2 rescues IRF4 expression

**Figure 6:**
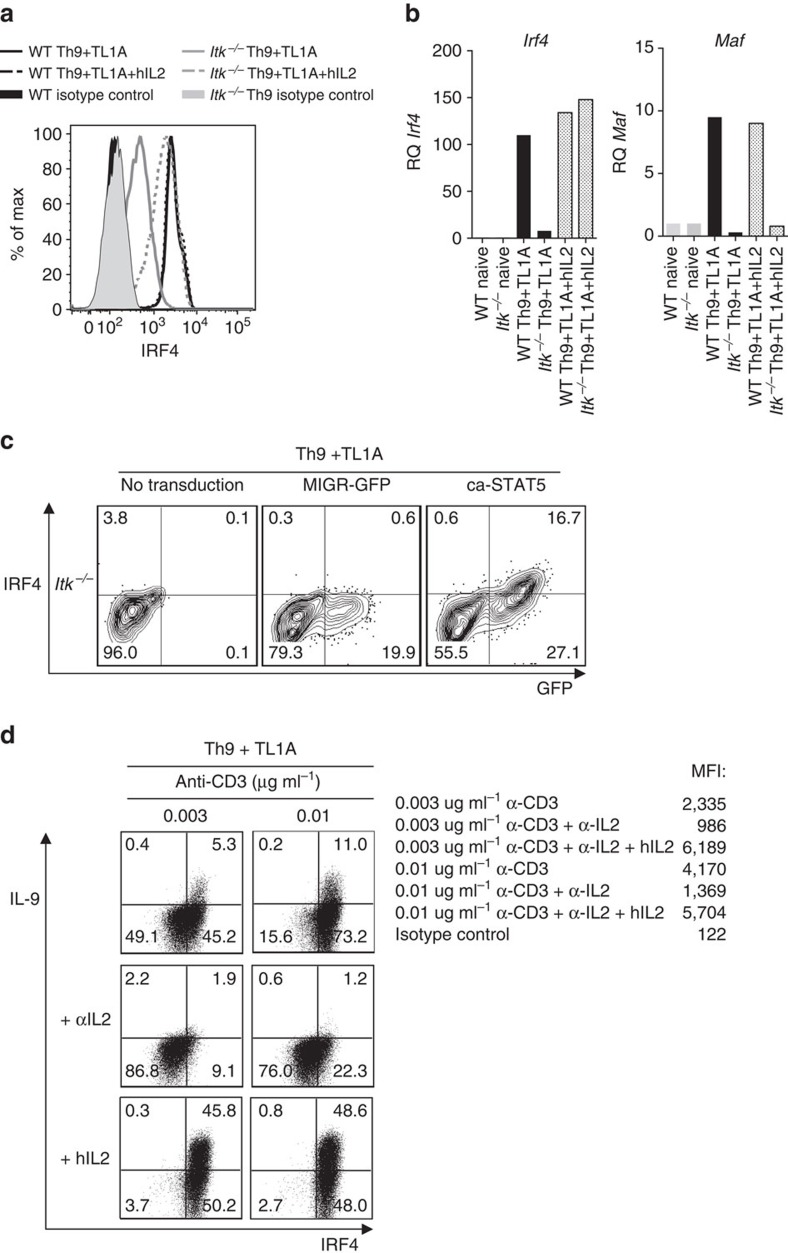
IL-2 rescues IRF4 expression in *Itk*^*−/−*^ CD4 T cells. (**a**) Sorted naive CD4^+^ T cells differentiated for 3 days under Th9 plus TL1A in absence or presence of blocking anti-IL-2 plus hIL-2, were stained for IRF4 and analysed by flow cytometry. (**b**) mRNA of *Irf4 and Maf* in cells differentiated as in **a** evaluated by qRT-PCR. (**c**) Itk-deficient CD4^+^ T cells were transduced with constitutively active STAT5 (ca-STAT5) or control retroviruses, differentiated under Th9 plus TL1A conditions and intracellular IRF4 determined. (**d**) Sorted naive WT CD4^+^ T cells were differentiated under Th9 plus TL1A with 0.003 or 0.01 μg ml^−1^ of anti-CD3, in absence or presence of blocking anti-IL-2 or blocking anti-IL-2 plus hIL-2, then restimulated with PMA and Ionomycin and IL-9 and IRF4 production were analysed by intracellular staining, MFI values for IRF4 are indicated. Data in figures **a**-**d** are representative examples from one of at least three independent experiments.

Strikingly, addition of hIL-2 not only rescued expression of IL-9, it also increased expression of IRF4 in Itk^−/−^ cells (Fig. 6a,b). In contrast, expression of *Maf*, was not rescued by IL-2 (Fig. 6b). Furthermore, IRF4 expression was also upregulated in response to retroviral transduction of ca-STAT5 (16.7% versus 0.3%; Fig. 6c). Previous data have shown that IL-2 facilitates activation and proliferation of weakly activated T cells stimulated with low affinity ligands^46^. Intriguingly, we observed that IL-2 increased the expression of IRF4 by WT cells stimulated with low concentrations of anti-CD3 (Fig. 6d). Conversely, treatment with anti-IL-2 antibody reduced expression of both IRF4 and IL-9. Although not all IRF4^+^ cells were pSTAT5^+^ (Supplementary Fig. 4), this may reflect a transient phosphorylation of STAT5. These results suggested that IL-2/STAT5-mediated pathways promote the induction of IRF4, at least under conditions of weak TCR stimulation.

### STAT5 binds to the IRF4 promoter

**Figure 7:**
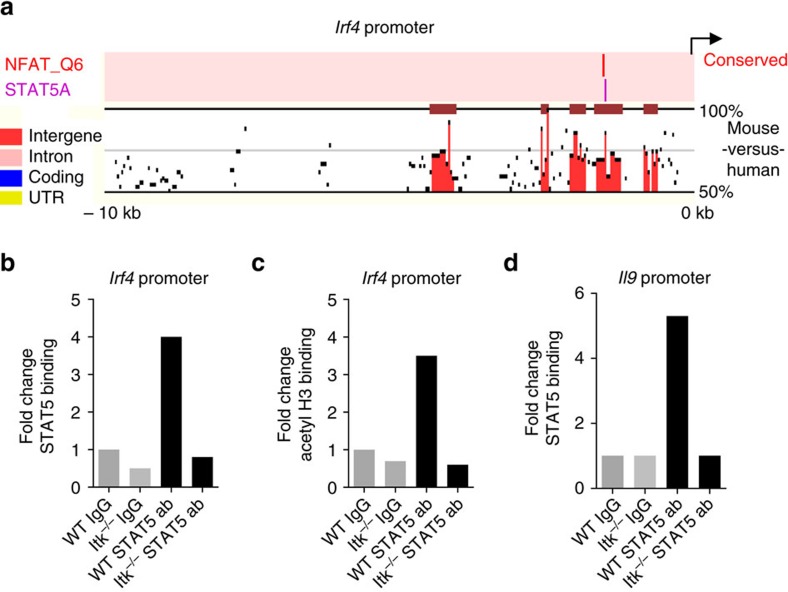
STAT5 binds to the IRF4 promoter. (**a**) A cross-species conserved STAT5-binding site in the promoter region of *Irf4* gene as predicted by the Mulan software at NCBI DCODE. (**b**,**c**) ChIP analysis of Th9 differentiated cells using anti-STAT5 (**b**), or anti-acetyl Histone H3 (**c**) antibodies and amplifying a region in the *Irf4* promoter ∼1.5 kb from the annotated first exon. (**d**) ChIP analysis using anti-STAT5 mouse antibodies were performed on *Il9* promoter. Data in **b**-**d** are representative of one out of two independent experiments.

To determine whether STAT5 binds directly to the *Irf4* promoter, we first examined the *Irf4* promoter sequences for conserved potential STAT5-binding sites. ENCODE software predicted a potential cross-species conserved STAT5-binding site 1.5 kb upstream of the first exon of *Irf4* (Fig. 7a). ChIP analyses using anti-STAT5 antibodies revealed direct association of STAT5 within this region in WT, but not Itk-deficient Th9 cells (Fig. 7b). Furthermore, evaluation of binding by acetylated histone H3 confirmed the active chromatin status of this region in WT but not Itk-deficient cells (Fig. 7c). Consistent with previous results showing binding of STAT5 to regions of the IL-9 promoter^33^,^43^,^44^, we also found STAT5-binding to a conserved site in the promoter region of the IL-9 gene in WT but not in Itk-deficient Th9 cells (Fig. 7d). Together, these data suggest that IL-2 and STAT5 are direct regulators of both *Il9* and *Irf4* expression, which are altered in the face of Itk deficiency.

### Itk deficiency protects against papain-induced lung disease

**Figure 8:**
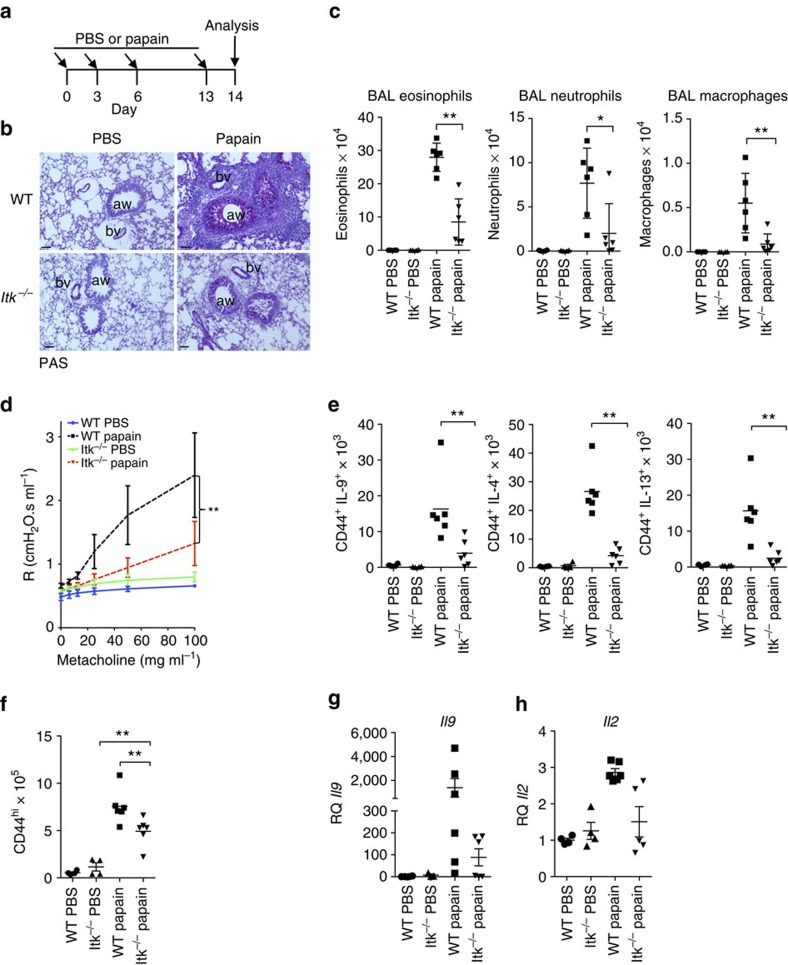
Itk-deficient mice are resistant to papain-induced lung inflammation. (**a**) Outline of papain-induced sensitization. (**b**) PAS-stained sections of lungs harvested at day 14 from WT and *Itk*^*−/−*^ mice challenged with papain or PBS, airways (aw) and blood vessels (bv) are marked. Scale bar, 50 μm. (**c**) Absolute numbers of macrophages, neutrophils and eosinophils in the BAL of mice treated with papain or PBS. (**d**) Airway resistance was measured in response to increasing doses of aerosolized methacholine in mice challenged with papain or PBS. **P<0.01 by two-way ANOVA. (**e**) Numbers of IL-9, IL-4 and IL-13–producing CD4^+^CD44^hi^ T cells in lungs are shown from cells harvested at day 14 from mice challenged with papain or PBS. (**f**) Numbers of activated (CD44^hi^) CD4^+^ cells in lungs after papain or PBS treatment. (**g**,**h**) mRNA levels of *Il9* (**g**) and *Il2* (**h**) measured by quantitative RT-PCR are shown for lung samples harvested on day 14 after initial challenge with papain or PBS. Values were normalized to the average level of *Il9* mRNA in the PBS-treated WT mice. Experiments were performed at least twice, using six female mice for papain-treated group and four female mice for PBS controls. Mean±s.e.m., *P<0.05, **P<0.01 using Mann–Whitney test (**c**,**e**,**f**).

Papain is an environmental protease that induces robust pulmonary allergic responses, including expression of IL-9. While initial responses to papain do not require T cells, but rather rely on ILC2 cells, the adaptive immune system is fully activated after 2 weeks of exposure, giving rise to T-cell expression of IL-9, in addition to IL-4 and IL-13 (refs 48, 49, 50). To determine whether Itk is required for T-cell IL-9 expression in the setting of a local allergic response *in vivo*, we exposed WT or *Itk*^*−/−*^ mice to 2 weeks of inhaled papain (Fig. 8a). Histological examination of lungs revealed that papain-treated *Itk*^*−/−*^ mice developed reduced immunopathology with decreased goblet cell hyperplasia and less cellular infiltration in the perivascular, interstitial and peribronchial areas compared with WT, similar to previous findings using an Ova challenge^30^ (Fig. 8b). Brochioalveolar lavage fluid from papain-sensitized *Itk*^*−/−*^ mice also contained reduced numbers of eosinophils, neutrophils and macrophages compared with WT (Fig. 8c). In addition, measurement of airway resistance in response to methacholine challenge revealed less airway hyper-reactivity in *Itk*^*−/−*^ mice, confirming their functional defects (Fig. 8d). Cellular analyses of lungs further showed that the numbers of IL-9-expressing infiltrating T cells were significantly reduced in *Itk*^*−/−*^ mice, as seen for IL-4 and IL-13-expressing T cells (Fig. 8e). Nonetheless, although Itk-deficient mice also had reduced numbers of T cells in the lungs, CD4^+^CD44^hi^ cells still expanded upon papain exposure, demonstrating that there was not a complete defect in T-cell responsiveness (Fig. 8f). *Il9* and *Il2* mRNA were also clearly reduced in the lungs of *Itk*^*−/−*^ mice 2 weeks post challenge (Fig. 8g,h). Thus, Itk deficiency also leads to Th9 defects *in vivo*.

### Itk-deficient mice have late defects in ILC2 function

**Figure 9:**
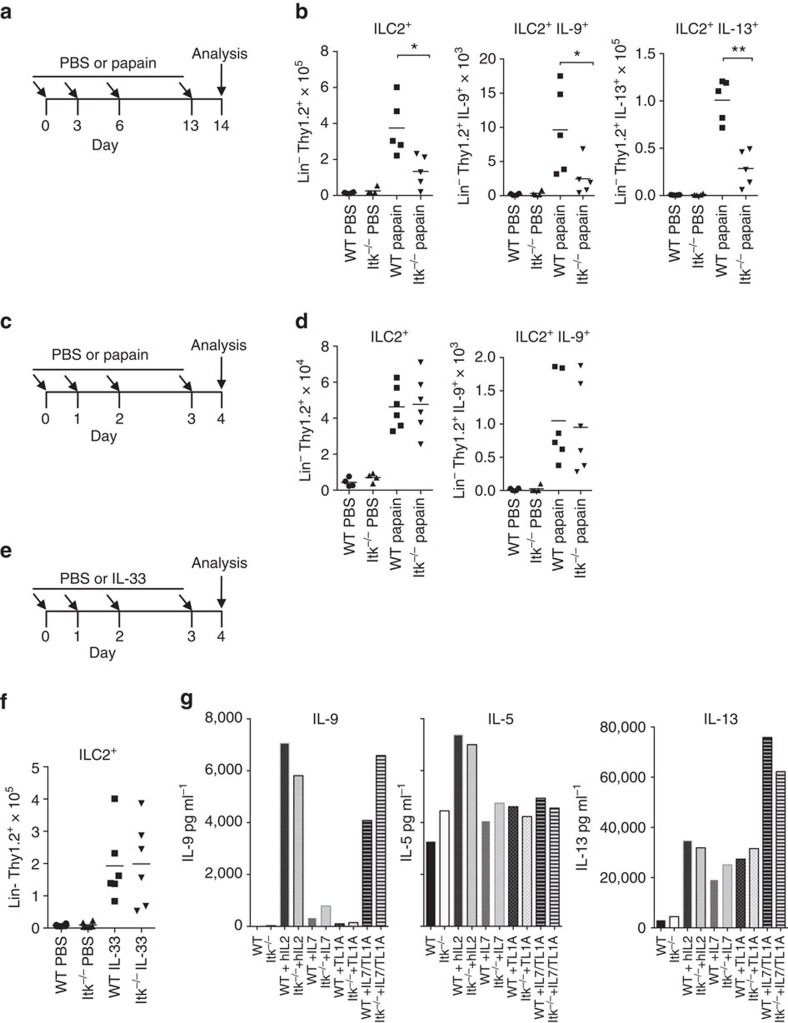
Itk-deficient mice have late defects in ILC2 function. (**a**) Outline of 14day papain-induced sensitization. (**b**) Numbers of ILC2^+^, IL-9- and IL-13-ILC2^+^ producing cells in lungs are shown from cells harvested at day 14 from female mice challenged with papain. (**c**) Outline of papain sensitization of female mice for 4 days. (**d**) Numbers of ILC2^+^ and IL-9-producing ILC2^+^ cells from mice treated with papain on day 4. (**e**) Outline of IL-33-induced sensitization for 4 days. (**f**) Numbers of ILC2^+^ cells in lungs harvested at day 4 from mice challenged with IL-33. (**g**) ILC2^+^ cells were sorted from IL-33-treated mice, cultured with 10 μg ml^−1^ of the indicated cytokines for 3 days and IL-9, IL-5 and IL-13 was measured in the culture supernatants (IL-9 and IL-5 have the same scale for Y axis). Experiments were performed at least twice using six female mice for papain-treated or IL-33 groups and four mice for PBS controls. (**b**,**d**,**f**) Mean±s.e.m. from one representative experiment, *P<0.05, **P<0.01 using Mann–Whitney test.

Another major source of IL-9 in response to papain challenge are cells of the ILC2 lineage, which are critical for the induction of type II T-cell responses in the lung^48^. Interestingly, ILC2 cells express Itk (ImmGen: http://www.immgen.org), despite their lack of TCR expression. To evaluate whether Itk deficiency affects ILC2s, we initially evaluated their numbers and IL-9 in mice treated repeatedly with papain for 2 weeks (Fig. 9a). Similar, to CD4^+^ T cells, we observed decreased numbers of ILC2, IL-9^+^ILC2 and IL-13^+^ILC2 cells 2 weeks post-sensitization with papain (Fig. 9b). Thus, Itk-deficient mice had decreased ILC2 responses at late times post-papain treatment.

Nonetheless, the appearance and function of ILC2 cells at late times post-papain challenge is dependent on IL-2 produced by T cells^50^,^51^, which is reduced in the lungs of papain-treated Itk-deficient mice (Fig. 8h). Thus, the ILC2 defect could be secondary to impaired T-cell function. To examine whether reduction of ILC2 cells is cell intrinsic, we evaluated their numbers 4 days after initial papain exposure (Fig. 9c). In contrast to the reduced number of ILC2 after 2 weeks of papain exposure, the expansion of ILC2 at early time points was not affected (Fig. 9d). In addition, IL-9 production by ILC2s was only minimally affected. Furthermore, ILC2 expansion by IL-33, a cytokine produced by epithelial cells that rapidly increases ILC2 numbers^52^ was not affected by Itk deficiency (Fig. 9e,f). *Ex vivo* ILC2 from IL-33-treated Itk-deficient mice secreted equivalent if not greater levels of IL-9, IL-5 and IL-13 cytokines in response to stimuli compared to WT ILC2s (Fig. 9g). Thus, Itk deficiency did not appear to lead to an intrinsic decrease in early ILC2 induction. These results suggest that the decreased ILC2 numbers and responses after 2 weeks of papain exposure in Itk-deficient mice may result from an indirect effect from reduced IL-2 production by T cells.

### ITK is required for Th9 differentiation in human cells

**Figure 10:**
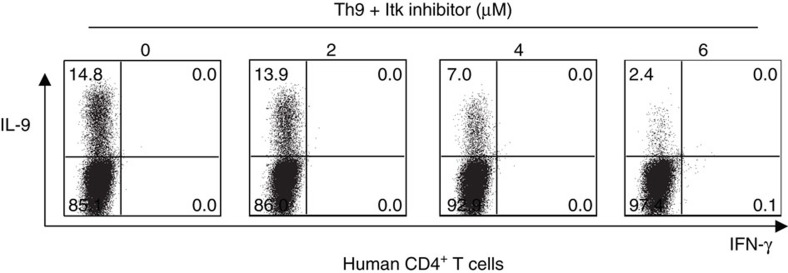
ITK is required for Th9 differentiation of human cells. Purified naive human CD4^+^ T cells were differentiated under Th9 conditions for 5 days in presence of increasing concentrations of ITK inhibitor. Cells were restimulated with PMA and Ionomycin and stained for IL-9. Data are representative of one of two independent experiments.

To determine whether ITK also influences IL-9 expression in human cells, naïve CD4^+^ T cells from human healthy donors were differentiated under Th9 conditions in the presence of an ITK inhibitor for 5 days. Similar to our observations in mouse T cells, the ITK inhibitor decreased expression of IL-9 in human T cells in a dose-dependent manner (Fig. 10). Thus, ITK is a positive regulator of IL-9 expression in both mouse and human T cells.

## Discussion

Itk is recognized as an important component of TCR signalling required for the development of Th2 pathology and expression of IL-4 in response to parasites and allergic asthma models. We demonstrate here that Itk also plays an important role in the expression of IL-9 and the induction of Th9 cells, a CD4^+^ T-cell subset relevant to asthma pathology. Using a papain hypersensitization model that induces both innate and adaptive allergic responses in the lung, we confirmed that Itk deficiency decreases pathological features of asthma, but also found evidence for decreased frequencies of both IL-9-producing T and ILC2 cells, despite normal early induction of ILC2s. Notably, when differentiated *in vitro* under Th9 conditions, *Itk*^*−/−*^ CD4^+^ T cells expressed virtually none of the signature cytokine IL-9. This phenotype is one of the most severe we have observed in *Itk*^*−/−*^ cells and was associated with reduced levels of pSTAT5, pS6 and IRF4, all of which could be rescued by IL-2. We further provide evidence for STAT5 binding to the promoters of both *Il9* and *Irf4*, which is lacking in the absence of Itk. In addition to confirming the importance of IL-2/STAT5-mediated pathways in the induction of *Il9*, our data reveal a novel role for Itk in promoting Th9 differentiation in both mouse and human and in the IL-2-mediated induction of *Irf4*, which encodes a critical transcription factor that links TCR signalling strength to downstream regulation of effector function.

IL-9 was first described as a cytokine that induced CD4^+^ T and mast cell proliferation. However, IL-9 also contributes to pathology in inflammatory bowel disease and autoimmune diseases, in addition to asthma models, where it was shown to induce mucus production, goblet cell metaplasia, expression of IL-13 and airway hypersensitivity^1^. Polymorphisms of the *IL9* gene have been linked to increased development of asthma, atopy and respiratory syncytial virus (RSV)-induced respiratory disease^53^. The involvement of IL-9 in multiple aspects of lung pathology suggests that it plays an important role in the pathogenesis of asthma and indeed, increased expression of IL-9 correlates with asthma severity in humans^3^,^4^,^5^,^6^. Data suggest ILC2 cells are an important early source of IL-13 and IL-9, which can induce Th2 differentiation^15^,^54^. In turn, IL-2 produced by T cells is important for later ILC2 expansion and IL-9 responses^50^,^51^. Although Th9 cells are not well appreciated, we find evidence for a discrete IL-9-producing T-cell population using a papain-induced model of airway hypersensitivity, that are affected by the loss of Itk. We further note, that decreased Th9 responses are observed despite normal early induction of ILC2 responses. Although we observe decreased numbers of ILC2 cells at later times post-papain sensitization, our data suggest that this reduction may result from impaired T-cell function and IL-2 production, highlighting the complex interactions between these cell types. Given the documented effects of IL-9 on asthma pathology, our findings suggest that defects in IL-9 production could be a critical part of Itk's effects on allergic asthma development.

Parallel to these findings, we observed a marked defect in IL-9 expression in Itk-deficient cells *in vitro*. Although IL-4 and TGFβ1 are the minimal requirements for Th9 differentiation in culture^16^,^17^,^18^,^19^; other cytokines, including the TNF family cytokine TL1A, can potentiate IL-9 expression^33^. However, even in presence of TL1A, Itk-deficient cells fail to express IL-9 and lack histone H3 acetylation on the *Il9* promoter, suggesting a global defect in opening of the *Il9* locus in Itk-deficient cells.

The activation of T cells via their TCR is critical for T cells to express cytokine receptors, effector cytokines, and other factors required for T-helper cell lineage differentiation^55^. Here, we show a direct correlation of the extent of TCR engagement and IL-9 expression in WT CD4^+^ T cells. Itk activity is required for TCR-induced nuclear translocation of NFATc1, which, in turn, is required for IL-9 expression^56^,^57^,^58^. Nonetheless, an activated NFATc1 construct was not sufficient to rescue IL-9 expression in Itk-deficient cells, despite its ability to rescue of IL-17A expression^26^. It is therefore of interest that, in CD8 cells, Itk and TCR signalling also play important roles in the induction of IRF4 (refs 23, 24, 59, 60). In CD8 cells, IRF4 is a key transcription factor that helps translate TCR signalling strength into metabolic and proliferative changes required for effector cell differentiation, proliferation and function. Itk is also required to induce metabolic changes necessary for effector cell differentiation and function in CD4 cells^27^ Interestingly, IRF4 is required for the differentiation of Th2, Th17 and Th9 cells^61^, all three of which are affected by Itk deficiency, suggesting that Itk is a critical component of pathways required to translate TCR signalling strength into functional outcomes required for effector T-helper cell function.

Under Th9 conditions, we observed a profound defect in IL-2 expression in Itk-deficient cells accompanied by decreased STAT5 phosphorylation and absent binding of STAT5 to the *Il9* promoter. Consistent with the critical role for IL-2 in IL-9 regulation^45^, IL-9 expression in Itk-deficient cells was completely rescued by addition of IL-2 or a ca-STAT5 construct. Notably, IL-2 and ca-STAT5 also rescued expression of IRF4. We further identified a cross-species conserved STAT5-binding site in the *Irf4* upstream region that bound STAT5 in ChIP analyses of WT, but not Itk-deficient cells. Although a previous study included *IRF4* among IL-2-induced genes found on microarrays^62^ and recent data implicate IL-2 as critical for nitrous oxide-induced IRF4 expression^63^, direct STAT5 binding of the IRF4 promoter has not been shown. Our studies suggest that IRF4 is a direct target of IL-2/STAT5 signalling and furthermore, that IL-2 is an important component of TCR-mediated induction of IRF4.

The observed decrease in STAT5 phosphorylation in Itk-deficient cells was somewhat surprising, as that we have previously found that Itk-deficient CD4^+^ cells show increased STAT5 phosphorylation under both Th17 and Treg-inducing conditions. Indeed, multiple studies have found an inverse correlation between IL-2-induced STAT5 phosphorylation and the extent of TCR engagement^27^,^47^,^64^,^65^. However, we found relatively low levels of IL-2 in supernatants from WT cells under Th9 conditions, and detected virtually no IL-2 in supernatants from Itk-deficient cells. In contrast, Th17 conditions induced much higher levels of IL-2, a somewhat surprising finding given that IL-2/STAT5 activation can be inhibitory for Th17 differentiation^66^. While this finding was unexpected, parallel observations have been made in Tfh cells, which are inhibited by IL-2 signalling, but paradoxically can express high levels of IL-2 (refs 67, 68). Our results further highlight that the differential activation of T cells via weak TCR activation or mutation of Itk can alter T-cell lineage differentiation both by affecting responses to and production of IL-2. Thus, at low TCR signals, there can be a paradoxical increase in STAT5 phosphorylation, as well as an uncoupling from PI3K-mediated pathways, perhaps secondary to changes in PTEN expression, as we have seen in the case of Itk deficiency, leading to changes in cell differentiation^27^. However, distinct alterations in differentiation can be observed under conditions where cells fail to produce IL-2 and induce CD25. Why Th9 conditions elicit such low levels of IL-2 remains unclear. A previous study suggested that TGFβ1 decreases T-cell proliferation and IL-2 production via inhibition of Itk^69^, yet our results suggest that these effects occur independent of Itk.

Moreover, IL-2's effects on IL-9 and IRF4 may not be limited to only STAT5-mediated pathways. Addition of IL-2 or ca-STAT5 boost expression of CD25, the high affinity IL-2R, potentiating the activation of multiple pathways downstream of IL-2. Indeed, addition of exogenous IL-2 to Itk-deficient Th9 cultures also greatly increased phosphorylation of S6, a downstream target of mTORC1, another regulator of metabolic activation affected by Itk-deficiency^27^. We also found that under Th9 conditions, IL-2 decreased *Pten* expression (data not shown), providing further insight into IL-2-mediated effects on T cells. IL-2, as a cytokine, has been shown to potentiate the activation and expansion of weakly activated T cells. Intriguingly, IL-4 (which is included in Th9 cultures) did not suffice in this function^46^. Our data provide new insight into the mechanisms by which IL-2 can augment signalling and potentiate T-cell activation in response to suboptimal TCR signals through the induction of IRF4 and mTOR.

In summary, we have uncovered a marked defect in the expression of IL-9 in CD4^+^ T cells deficient for Itk, which is linked to impaired IL-2 expression and which affects both T cells and ILC2 cells in the lung. Our findings therefore suggest that Itk contributes to the regulation of multiple cytokines and cells involved in Type II-mediated immunity and pathology, and that inhibition of Itk may help prevent the differentiation of T cells into pro-allergic effector cells. Nonetheless, the effects of Itk inhibition may be complex, as that recent data suggest that Itk inhibitors can exacerbate asthma in an animal model, perhaps due to decreased T-cell apoptosis^70^. We also note that loss of Itk greatly increases T-cell sensitivity to IL-2. Thus, whether inhibiting Itk or other TCR signalling components will be a useful therapeutic strategy for treating or preventing symptoms of asthma and other diseases in which IL-9 participates remains an important question.

## Methods

### Mice

*Itk*^*−/−*^ (ref. 34) and WT mice were backcrossed 12 generations onto the C57BL/6 background. CD45.1 congenic C57BL/6 mice were purchased from Taconic. All mice used were between 7 and 10 weeks old. For *in vitro* experiments, either male or female mice were used. For papain and IL-33 sensitization, only female mice were used. Animal husbandry and experiments were performed in accordance with approved protocols by the National Human Genome Research Institute's Animal Use and Care Committee, NIH.

### Mouse T-cell purification and culture

CD4^+^ T cells were purified by negative selection from pooled lymph nodes and spleens from 6 to 12 age and sex matched male or female mice, using a magnetic cell separation system according to the manufacturer's protocol (Miltenyi Biotec). Naive (CD4^+^ (PerCP/Cy5.5, clone RM4-5) CD44^low^ (APC, clone IM7) CD62L^hi^ (eFluor 450, clone MEL-14) CD25^neg^ (PE, clone PC61.5) T cells) were purified by cell sorting to greater than 99% purity (Antibodies were purchased from eBioscience). Cells were cultured in complete IMDM media as indicated^26^. Briefly, sorted naive CD4^+^ T cells (2 × 10^5^) were co-cultured at a ratio of 1:5 with mitomycin-treated T-depleted splenocytes as APCs in 48-well plates under Th9 conditions (1 μg ml^−1^ anti-CD3 (clone 2C11), 3 μg ml^−1^ anti-CD28 (clone 37.51), plus 20 ng ml^−1^ IL-4 (catalogue number 214-14, PeproTech), 5 ng ml^−1^ TGFβ1 (catalogue number 100-21, PeproTech) and 10 μg ml^−1^ anti-IFN-γ (clone XMG1.2) with or without 10 ng ml^−1^ mTL1A (catalogue number 1896TL, R&D) for 3 days or as indicated in figure legends. Th17 conditions included 1 μg ml^−1^ anti-CD3, 3 μg ml^−1^ anti-CD28 plus 20 ng ml^−1^ of IL-6 (catalogue number 216-16, PeproTech), 5 ng ml^−1^ of TGFβ1, and 10 μg ml^−1^ of each anti-IL-4 (clone 11B11), anti-IFN-γ, and anti-IL-12 (clone C17.8) antibodies. Th2 conditions included 1 μg ml^−1^ anti-CD3, 3 μg ml^−1^ anti-CD28, 20 ng ml^−1^ IL-4 and 10 μg ml^−1^ anti-IL-12. Antibodies were purchased from BioXcell unless otherwise indicated. For inhibition studies, sorted naive CD4^+^ T WT cells and APCs were incubated either with an Itk inhibitor (BMS-509744, Calbiochem) or DMSO vehicle for 45 min before stimulation with the indicated differentiation conditions for 3 days. For evaluating responses to IL-2, sorted naive CD4^+^ T cells from WT and *Itk*^*−/−*^ mice were stimulated under Th9 or Th9 plus TL1A conditions, in the absence or presence of blocking anti-IL-2 (10 μg ml^−1^; clone JES6-1A12, BioXcell) plus hIL-2 (100 U ml^−1^) (catalogue number 136, NIH AIDS Reagent Program). To study the role of TCR stimulation, naïve CD4^+^ T WT cells were differentiated with varying concentrations of anti-CD3 (0.003, 0.01, 0.1 or 1 μg ml^−1^) under Th9 plus TL1A conditions.

### Human CD4^+^ T-cell purification and culture

Human peripheral blood mononuclear cells were obtained from the NIH Department of Transfusion Medicine under NIH Clinical Center IRB-approved protocol 99-CC-0168 ‘Collection and Distribution of Blood Components from Healthy Donors for *In Vitro* Research Use' Naive CD4^+^ T cells were isolated from PBMCs by negative selection (catalogue number 130-094-131, Miltenyi). Stimulation under Th9 differentiation conditions was performed using Human T-activator CD3/CD28 Dynabeads (catalogue number 11131D, Invitrogen) with human IL-4 (20 ng ml^−1^) and human TGFβ1 (5 ng ml^−1^). Differentiated cells were split into new media with cytokines on day 3, on day 5 cells were restimulated with PMA and Ionomycin for 4 h and intracellular IL-9 levels were determined by flow cytometry using anti-human IL-9 antibodies (PE, MH9A4, BioLegend). To test the ITK inhibitor, naive CD4^+^ T cells were preincubated with the inhibitor for 45 min prior to addition of the Th9 cocktail.

### Retrovirus production and infection

MIGR (IRES-GFP)-based plasmids expressing ca-NFATc1 (gift of N. Clipstone), ca-STAT5 (gift of Jinfang Zhu) and ITK (gift of L. Berg; 12.5 μg) were transfected into 293 T cells with Fugene (Roche). After 48 hr, retroviral supernatants were collected. Sorted naïve *Itk*^*−/−*^ CD4^+^ T cells from *Itk*^*−/−*^ mice in presence of APCs were activated under Th9 conditions (1 μg ml^−1^ anti-CD3, 3 μg ml^−1^ anti-CD28, 20 ng ml^−1^ IL-4, 5 ng TGFβ1) in presence of T-depleted splenocytes as APCs for 36 hr. Retrovirus supernatants were added to the cells and spun at 2,500 r.p.m. for 1.5 h at room temperature with 8 μg ml^−1^ of polybrene (Sigma). After 24 hr, infected cells were differentiated under Th9 conditions (1 μg ml^−1^ anti-CD3, 3 μg ml^−1^ anti-CD28, 20 ng ml^−1^ IL-4, 5 ng ml^−1^ TGFβ1, 10 ng ml^−1^ TL1A) for 72 h and restimulated with PMA+ionomycin and stained for intracellular IL-9 (PE, RM9A4, BioLegend) and IRF4 (eFluor 450, clone 3E4, eBioscience).

### Flow cytometric analyses

For cytokine determination, differentiated cells were stimulated for 4 h with 2 ng ml^−1^ of PMA (Sigma) and 1 μg ml^−1^ of ionomycin (Sigma) in presence of Golgi stop (BD) and stained for CD4^+^ (PE-Cy7, clone RM4-5); or intracellular staining for IL-9 (PE, RM9A4, BioLegend), IL1-7A (APC, clone eBio17B7), IFN-γ (PerCP/Cy5.5, clone XMG1.2) was performed according to the manufacturer's instruction (eBiosciences). For intracellular levels of transcription factors, CD4^+^ T cells were fixed with 4% PFA and permeabilized with methanol at -20 C and stained with phospho-STAT5 (PE, clone 47/Stat5(pY694)), pSTAT6 (APC, clone 18/pStat6, both antibodies from BD Biosciences) and pS6 (Alexa Fluor 488, clone D68F8, Cell Signaling). In some experiments, cells were also stained for CD25 (PE, clone PC61.5). Cells were stained with indicated antibodies for 60 min in the dark at 4 °C. Analyses were performed by using a FlowJo software (Tree Star).

### Cytokine measurement

Supernatants from CD4^+^ T-cell differentiation cultures were collected at day 3 before restimulation with PMA+ionomycin and analysed for cytokines by a multiplex, bead-based protein detection assay. Supernatants from *ex vivo*-stimulated ILC2 cultures were collected at day 3. Supernatants were probed for IL-9 (catalogue number 171G5008M), IL-2 (catalogue number 171G5003M), IL-17A (catalogue number 171G5013M), IL-5 (catalogue number 171G5006M), IL-13 (catalogue number 171G5012M using BioRad's Bio-Plex Pro Mouse Cytokine Assay according to the manufacturer's protocol.

### RNA isolation and analysis

Total RNA was isolated from the differentiated cells at different time points with the RNeasy Mini Kit (QIAGEN) and was reverse transcribed with random hexamer primers and M-MLV Reverse Transcriptase (Applied Biosystems). Quantitative RT-PCR was performed on Step One Plus Real-Time PCR System (Applied Biosystems) using TaqMan assays (Applied Biosystems) for mRNAs indicated in the figures. The samples were normalized to 18S RNA and data expressed as relative to WT naïve levels using the 2^-ΔΔCT^ method.

### Chromatin immunoprecipitation

Detection of STAT5 binding and histone H3 acetylation of the *Irf4* promoter was performed by using a commercial ChIP kit (catalogue number 17-245, Upstate Biotechnology) according to the manufacturer's instructions. Th9 differentiated cells from WT and Itk-deficient mice were treated as specified in the kit's protocol. Binding of transcription factors to the *Irf4* and *Il9* promoters region in Th9 CD4^+^ T cells was characterized by ChIP using anti-STAT5 (catalogue number sc-74442 X, Santa Cruz) 5 μg per 1 × 10^6^ cells, or anti-acetyl histone H3 (catalogue number 06-599B, Millipore) 5 μg per 1 × 10^6^ cells. PCR detection (Sybergreen Invitrogen TaqDNA polymerase) of the immunoprecipitates was performed using the following primers: *Irf4* gene promoter region: 5′ CTCTGACAATGGAAAACTAATTG -3′ (forward) and 5′- GAAAACTGGTGCAGAGATGC -3′ (reverse); *Il9* promoter: 5′- TTTTCCTTTAGTATTTCAGAACCC -3′ (forward) and 5′- AAAATCAGTCTGAGTCACTTGAC -3′ (reverse). Input controls, representing the starting material before the immunoprecipitation step, were included in the PCR, alongside with the immunoprecipitated samples and Ab-negative control samples.

### Papain-induced lung inflammation

For papain-induced lung inflammation, aged-matched female mice between 8 and 10 weeks were anesthetized with isoflurane and exposed intranasally to PBS or 25 μg papain (Calbiochem) in 30 μl PBS on days 0, 3, 6 and 13; or on days 0, 1, 2 and 3. Around 12–16 h after the last challenge, BAL fluid was obtained by direct cannulation of the lungs with a 20-gauge i.v. catheter and lavage with 500 μl 1% FBS in PBS (for cytokine analysis) and with 750 μl 1% FBS in PBS. Samples for cellular analysis were prepared by pooling the two lavages fraction, and a portion was used to determine the total cell counts. Cells from the BAL were stained using Abs against CD45.2 (APC-eF780, clone 104), Siglec F (APC, clone 1A5, Miltenyi), F4/80 (PE-Cy7, clone BM8), Ly-6G (PerCP/Cy5.5, clone RB6-8C5) and CD11b (FITC, clone M1/70; all antibodies were purchased from eBioscience except Siglec F). Eosinophils were identified as CD45.2^+^ F4/80^−^ Ly 6G^-^ CD11b^+^ SiglecF^+^. Lungs for histology were fixed in 10% formalin, embedded in paraffin sectioned and stained with periodic acid-schiff (PAS) for detection of goblet cells and mucus production. For cytokine analyses, cells were isolated by incubating lung fragments with 100 U collagenase for 1 h, thereafter the isolated cells were restimulated with PMA/ionomycin for 4 h and stained with LIVE/DEAD Fixable Blue Dead Cell Stain (catalogue number L34957, Life Technologies) and the antibodies for: TCRβ (APC-eFluor780, clone H57-597), CD45.2 (PE-Cy7, clone 104), CD44 (Alexa Fluor 700, clone IM7), IL-13 (eFluor 660, clone eBio13A), IL-4 (FITC, clone BVD6-24G2) (antibodies purchased from eBioscience); CD4 (V500, clone RM4-5, BD Biosciences), IL-9 (PE, RM9A4, BioLegend). For RNA extraction, lung tissue was stored in RNA*later* (ThermoFisher) and homogenized with a Precellys 24 (Bertin Laboratories) in lysis buffer and purified with the PureLink RNA Mini Kit (Life Technologies). mRNA expression for *Il9* and *Il2* was evaluated using iTaq Universal probes One-Step Kit (BioRad) and using *Il9* and *Il2* TaqMan probes from Life Technologies.

### Expansion of ILC2 by IL-33

For IL-33-mediated ILC2 expansion, age-matched female mice between 8 and 10 weeks of age were anesthetized with isoflurane and exposed intranasally to 1 μg IL-33 (catalogue number 580506, BioLegend) in 30 μl of PBS on days 0, 1, 2 and 3. Control mice received only PBS. About 16 h later lungs were isolated for analysis as described in the previous section.

### Isolation and culture of ILC2 cells

Age-matched female mice between 8 and 10 weeks old received intranasally 0.5 μg of IL-33 in 30 μl of PBS at days 0, 1, 2 and 3. Lungs were harvested and treated as described above. ILC2 were sorted by gating on the live CD45.2^+^ (PE-Cy7, clone 104) cells and then for the absence of lineage markers of TCRβ (clone H57-597) TCRγδ (clone, eBioGL3), CD4 (clone RM4-5), CD8 (clone 53-6.7), NK1.1 (clone PK136), CD11c (clone N418), CD11b (clone M1/70), CD19 (clone eBio1D3), Ly-6G (clone RB6-8C5) (all lineage marker antibodies were labelled with FITC) but expression of Thy 1.2 (eFluor 450, clone 53-2.1, BioLegend) and ICOS (PerCP/Cy5.5, clone C398.4, BioLegend). *Ex vivo* sorted ILC2 cells were cultured at 50,000 cells per well for 72 h with either IL-2, IL-7 (catalogue number 217-17, PeproTech) or mTL1A and IL-5, IL-9, IL-13 were determined in supernatants as described above.

### Measurement of allergic airway reactivity

Bronchial reactivity was determined 12 h after the last challenge of papain. Mice were anesthetized by i.p. administration of ketamine/xylazine mixture (1 ml ketamine (100 mg ml^−1^), 0.5 ml xylazine (20 mg ml^−1^), and 8.5 ml PBS). An 18-gauge blunt-end needle was inserted into the trachea, and the animals then were ventilated mechanically. Baseline measurements were recorded after the aerosol administration of saline, followed by doubling doses of methacholine (6.25–100 mg ml^−1^) using flexiVent (Scireq Scientific Respiratory Equipment).

### Statistical analyses

Results were expressed as the mean±s.e.m. Statistical differences between the analysed groups were calculated with the paired Student's *t*-test. Values of *P*<0.05 are considered significant. Alternatively, for evaluation of Papain- or IL-33 sensitization, groups were compared using Mann–Whitney tests. The size of groups was determined empirically for showing adequate statistical evaluation of differences in responses to papain and IL-33. For methacholine resistance studies, groups were analysed using Two-Way ANOVA. Graphs were done in Prism (GraphPad).

## Additional information

**How to cite this article:** Gomez-Rodriguez, J. *et al*. Itk is required for Th9 differentiation via TCR-mediated induction of IL-2 and IRF4. *Nat. Commun.* 7:10857 doi: 10.1038/ncomms10857 (2016).

## Supplementary Material

Supplementary Information Supplementary Figures 1-4
