# Supplementary material for: Itk is required for Th9 differentiation via TCR-mediated induction of IL-2 and IRF4
Source: Nat Commun. 2016 Mar 3;7:10857. doi: 10.1038/ncomms10857 (PMC4782063; doi:10.1038/ncomms10857)
Supplement: Supplementary Information — Supplementary Figures 1-4 [file ncomms10857-s1.pdf]

Gomez-Rodriguez et al. Supplementary Figures

Supplementary Figure 1

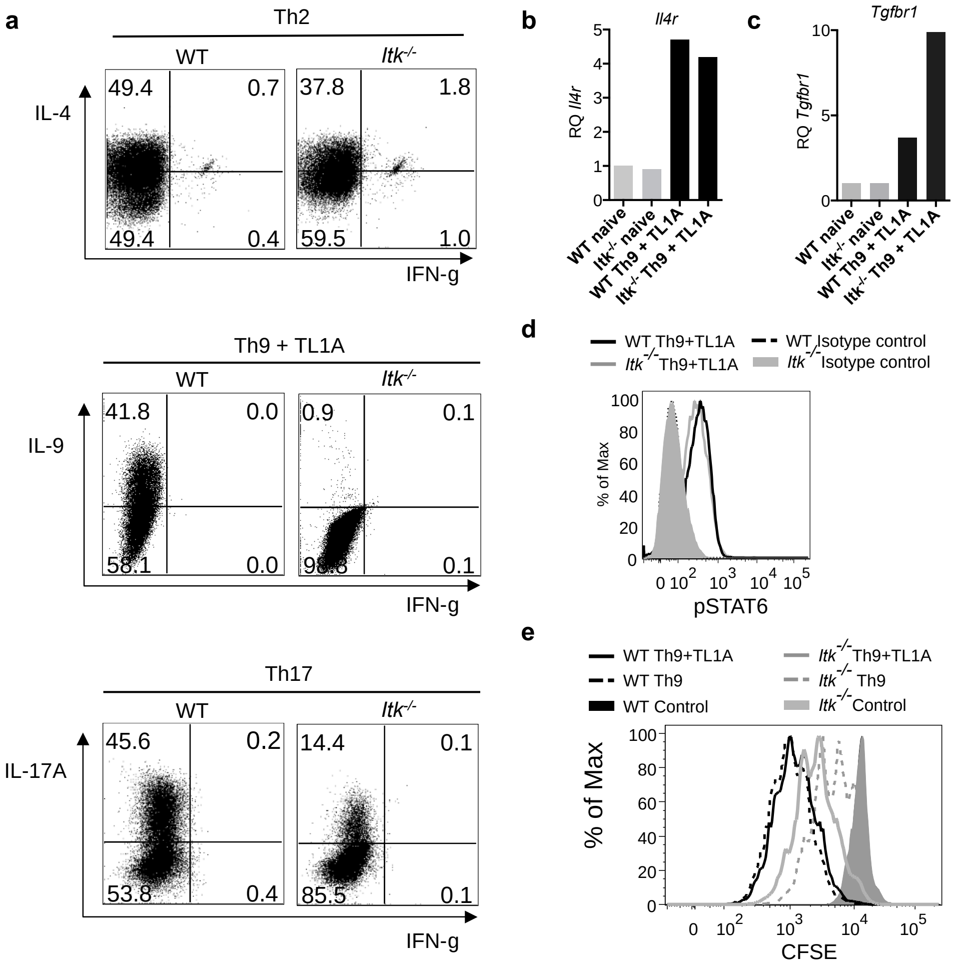

Supplementary Fig. 1. Itk is required for Th9 differentiation. (**a**) Sorted naïve CD4+ T cells from WT and *Itk-/-* mice were differentiated under Th2, Th9 plus TL1A, or Th17 conditions, cells were restimulated with PMA and Ionomycin and IL-4, IL9, IL17A and IFNγ were evaluated by intracellular staining. (**b,c**) mRNA for *Il4r* (**b**) and *Tgfbr1* (**c**) was determined by qRT-PCR from CD4 T cells from WT and *Itk-/-* mice differentiated under Th9 plus TL1A conditions. (**d**) Sorted naïve CD4+ T cells from WT and *Itk-/-* mice were differentiated under Th9 plus TL1A conditions for 3 days and pSTAT6 was determined by flow cytometry: WT (black), *Itk-/-* (grey) lines. (**e**) Sorted naïve CD4+ T cells from WT and *Itk-/-* mice were stained with CSFE and differentiated under Th9 plus TL1A conditions, WT (black), *Itk-/-* (grey) lines. Full grey (*Itk-/-*) or black (WT) curves correspond to non-activated labeled cells. Cells were restimulated with PMA and Ionomycin and stained for IL-9 and IFN-γ. Results in (**a-e**) are representative of one out of at least 3 independent experiments.

Supplementary Figure 2

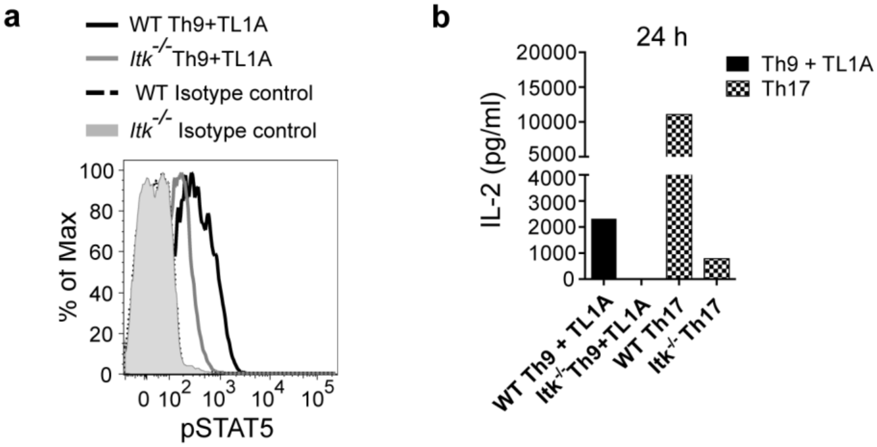

Supplementary Fig. 2. Defects in IL-2 signaling in Itk-deficient cells. (**a**) Sorted naïve CD4^{+} T cells from WT and *Itk*$^{-/-}$ mice were differentiated under Th9 plus TL1A conditions and pSTAT5 levels were determined by flow cytometry at 24h: WT (black), *Itk*$^{-/-}$ (grey) lines. (**b**) Sorted naïve CD4^{+} T cells from WT and *Itk*$^{-/-}$ mice were differentiated as in (**a**) or under Th17 conditions and IL-2 levels were determined in supernatants at 24h by Luminex. Results in (**a,b**) are representative of one out of at least 3 independent experiments.

Supplementary Figure 3

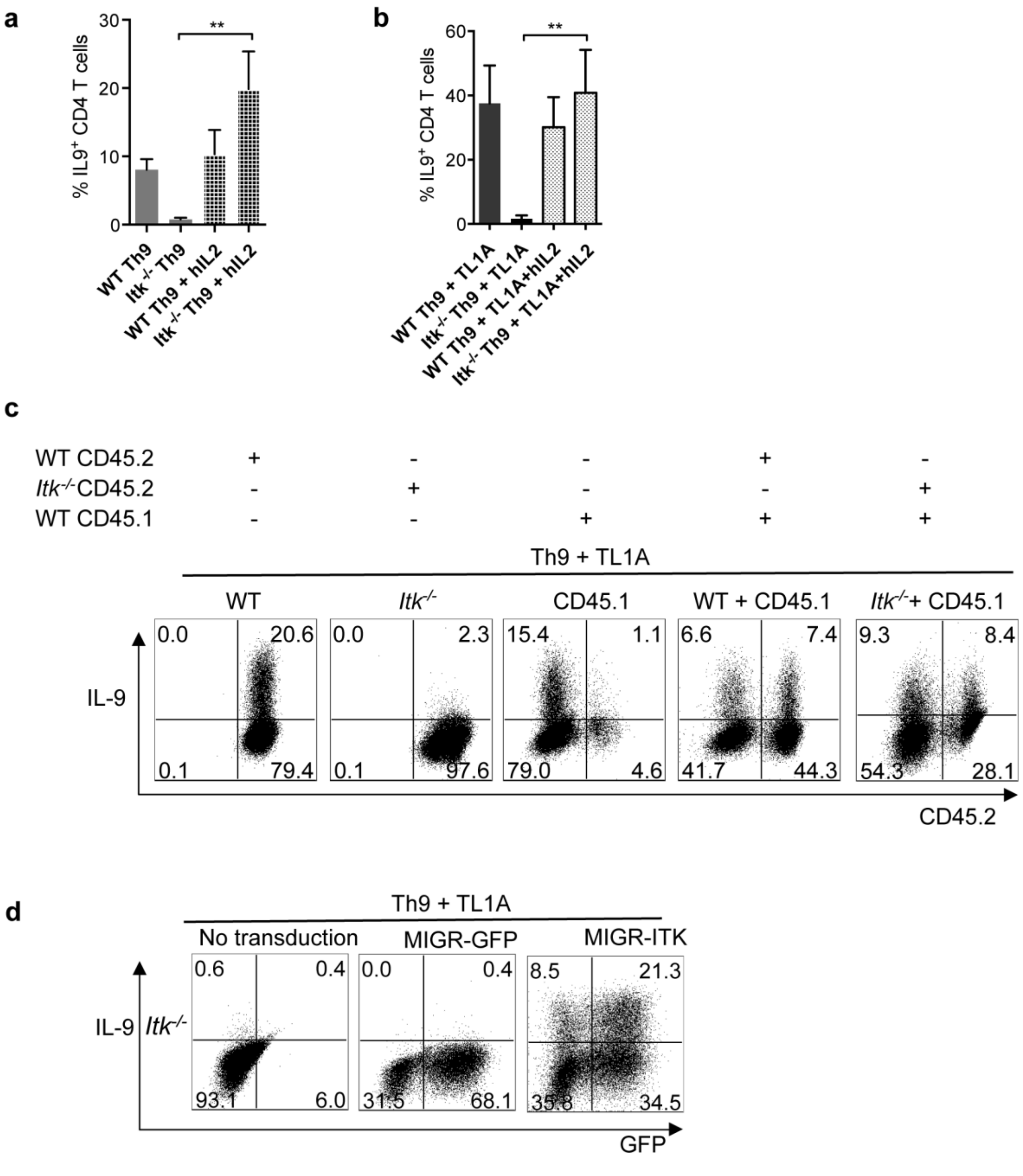

Supplementary Fig. 3. IL-2 rescues the defect in Th9 differentiation in Itk-deficient cells. (**a,b**) Sorted naïve CD4^{+} T cells from WT and *Itk*$^{-/-}$ mice were differentiated under Th9 (**a**) or Th9 plus TL1A (**b**) conditions in absence or presence of anti-murine IL-2 plus hIL2. IL-9 production was determined by intracellular staining after PMA and Ionomycin restimulation. The panels show means ± s.e.m. of IL-9^{+} CD4^{+} T cells from 5 independent experiments, ** p<

0.01, determined by two-tailed unpaired Student's t-test. (**c**) Sorted naïve CD4$^+$ T cells from WT or *Itk*$^{-/-}$ CD45.2 mice were cultured either alone (left two plots) or with naïve CD4$^+$ T cells from CD45.1 mice (right two plots) plus WT CD45.2 APCs and differentiated for 3 days under Th9 conditions plus TL1A as indicated in the Table. Cells were restimulated with PMA and Ionomycin and IL-9 determined by flow cytometry. CD45.2 staining cells in the CD45.1 alone sample are presumed to be derived from contamination from the WT CD45.2 APC population. (**d**) IL-9 production by *Itk*$^{-/-}$ is rescued by re-expression of Itk. Itk-deficient CD4$^+$ T cells were transduced with the indicated retroviruses, differentiated under Th9 plus TL1A and IL-9 production was determined by intracellular staining after PMA and Ionomycin restimulation. Data in (**c,d**) are representative of one out of 3 different experiments.

Supplementary Figure 4

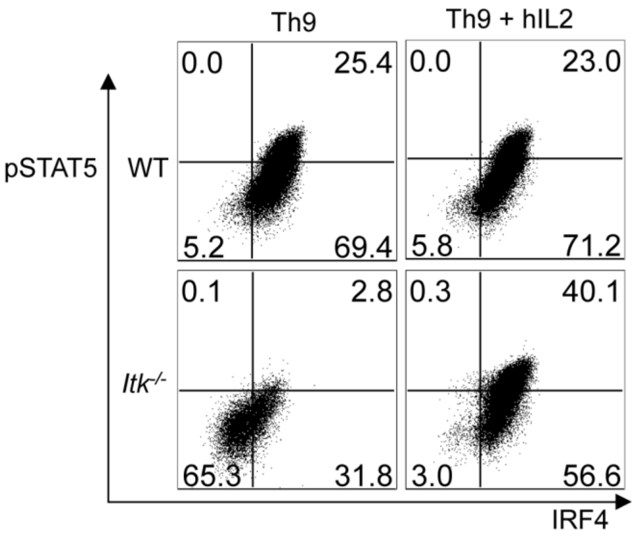

Supplemental Fig. 4. IL2 rescues pSTAT5 expression under weak TCR signaling. Sorted naïve $CD4^+$ T cells from WT and *Itk*$^{-/-}$ mice were differentiated under Th9 conditions in absence or presence of anti-murine IL2 plus hIL-2 and analyzed for pSTAT5 and IRF4 production by intracellular staining. Data are representative of one out of 3 independent experiments.
